# LRRC59 serves as a novel biomarker for predicting the progression and prognosis of bladder cancer

**DOI:** 10.1002/cam4.6542

**Published:** 2023-09-14

**Authors:** Peng Zhang, Xiaodu Xie, Chunming Li, Chaohua Zhang, Peihe Liang

**Affiliations:** ^1^ Department of Urology The Second Affiliated Hospital of Chongqing Medical University Chongqing People's Republic of China; ^2^ Department of Hepatobiliary Surgery The Second Affiliated Hospital of Chongqing Medical University Chongqing People's Republic of China; ^3^ Department of Gastrointestinal Surgery The Second Affiliated Hospital of Chongqing Medical University Chongqing People's Republic of China

**Keywords:** bioinformatics, bladder cancer, cell proliferation, LRRC59, migration, prognosis

## Abstract

**Background:**

Leucine‐rich repeat‐containing protein 59 (LRRC59) is an endoplasmic reticulum membrane protein involved in various cancers, but its role in bladder cancer (BC) has not been reported. The aim of the present study was to investigate the role of LRRC59 protein in BC progression and prognosis.

**Methods:**

The expression profile and clinical significance were retrieved from BC patients in the Cancer Genome Atlas database. The methylation status of LRRC59 was analyzed by UALCAN and MethSurv databases. Potential signaling pathways and biological functions were explored by functional enrichment analysis. Immunocyte infiltration was evaluated by CIBERSORT analysis. The prognostic value of LRRC59 was evaluated by Kaplan–Meier and Cox regression analyses. Overall survival (OS) was predicted by the nomogram plot established in this study. LRRC59 expression in 10 pairs BC and adjacent noncancerous tissues were analyzed by immunohistochemistry (IHC). Cell proliferation, migration, and invasion were detected by CCK8, colony formation assay, transwell assay, and cell scratch assay, respectively. Proteins related to epithelial–mesenchymal transition and apoptosis were detected by western blot.

**Results:**

LRRC59 overexpression significantly decreased OS, disease‐specific survival, and progress‐free interval of BC patients. LRRC59 was a prognostic marker for OS and its hypomethylation status signified a poor prognosis. LRRC59 overexpression was correlated with infiltration of resting memory CD4 T cells, memory activated CD4 T cells, resting NK cells, macrophages M0, M1, M2, and neutrophils. IHC showed that the LRRC59 expression in BC tissue was significantly higher than that in adjacent noncancerous tissue. Knockdown of LRRC59 expression inhibited the proliferation of BC cells and reduced their migratory ability. Western blot showed that Snail and vimentin protein expressions decreased, while E‐cadherin expressions increased.

**Conclusions:**

LRRC59 expression can predict the outcome of BC independently and serve as a new biomarker for diagnosis.

## 1 INTRODUCTION

Bladder cancer (BC) is one of the common malignant tumors of the urinary system with high morbidity and mortality.^1^ According to the data released by the WHO International Agency for Research on Cancer in 2020, the morbidity and mortality of BC have been on the rise yearly, ranking second only to prostate cancer in male genitourinary tumors.^1^, ^2^ More than 95% bladder tumors belong to bladder urothelial carcinoma. Despite the continuous emergence of new medications such as immune checkpoint inhibitors (ICIs) in recent years, the prognosis of BC remains unchanged significantly.^1^, ^2^, ^3^ It is therefore urgent to identify new targets for early diagnosis, treatment, and prognostic prediction of BC.

Leucine‐rich repeat‐containing protein 59 (LRRC59) is a tail‐anchored endoplasmic reticulum (ER) membrane protein consisting of a small C‐terminal domain facing the ER lumen, a leucine‐rich repeat (LRR), and a coiling domain facing the cytoplasm.^4^, ^5^ LRRC59 has been shown to be a binding transporter of the cellular inhibitor PP2A (CIP2A) in prostate cancer,^6^ and its fusion transcripts are present in various malignancies such as ovarian, esophageal, and prostate cancers.^7^, ^8^ LRRC59 was found to associate with TNM stage, lymph node metastasis (LNM), histological differentiation, and poor prognosis of lung adenocarcinoma,^9^ but its role in BC has not been clearly elucidated.

The aim of this study was to clarify the clinicopathologic characteristics of BC and explore the expression profile of LRRC59, its prognostic significance and role in BC tumor cell proliferation and metastasis.

## 2 MATERIALS AND METHODS

### 2.1 Collection of data

The mRNA expression patterns and clinical data of 408 BC patients were retrieved from the Cancer Genome Atlas (TCGA) database (http://portal.gbc.cancer.gov/), the largest database of cancer genetic information including 33 cancer types based on large‐scale genome sequencing as well as genomic, transcriptomic, epigenetic, proteomic, and other omics data. Additional prognostic data were obtained from Liu's study.^10^

### 2.2 Tissue samples and cell lines

From October 2020 to September 2021, tissue samples were obtained from 10 BC patients who underwent surgery at the Second Affiliated Hospital of Chongqing Medical University (Chongqing, China). All these patients were newly diagnosed patients without previously receiving any radiotherapy, chemotherapy, or interventional therapy. According to the results of postoperative pathological examination, WHO 2004 grade was high in six cases and low in four cases, using the adjacent tissues as the control. This study protocol was approved by the Ethic Committee of the said University (No. 128, 2022). Human bladder epithelial immortalized SV‐HUC‐1 cells, and human BC T24, 5637, and J82 cells were provided and maintained by the Immunological Complement Laboratory of the Army Military Medical University (Chongqing, China).

### 2.3 Reagents

The reagents used in this study were Hams F‐12K medium (Wuhan Servicebio), RPMI 1640 medium, and FBS (Gibco). The three groups of LRRC59 interference experimental plasmids with different sequences and one negative control plasmid (Chongqing Baoguang Biotechnology Co., Ltd.) are as follows:

shLRRC59#1:5′‐CCTGGATCTGTCTTGTAATAA‐3′

shLRRC59#2:5′‐GTAATAAACTGACTACTCT‐3′

shLRRC59#3:5′‐GCAGTGTAAGCAGTGTGCAAA‐3′

shLRRC59#NC:5′‐CCTAAGGTTAAGTCGCCCTCG‐3′

Other reagents and instruments included the transfection reagent Lipofectamine3000 (Invitrogen); RNA extraction TRIzol Kit, RNA reverse transcription kit, and quantitative detection kit (TaKaRa); qRT‐PCR primers (Shanghai Sangon Biotech Co., Ltd.); CCK‐8 kit (Chongqing Baoguang Biotechnology Co., Ltd.); transwell cell chamber (Corning); Matrigel (BD); crystal violet staining solution (Chongqing Baoguang Biotechnology Co., Ltd.); rabbit anti‐human LRRC59 polyclonal antibody (ab184143, Abcam); rabbit anti‐human GAPDH monoclonal antibody (2118), and mouse anti‐human β‐actin monoclonal antibody (3700; Cell Signaling Technology); rabbit anti‐human E‐cadherin polyclonal antibody (BS72286), rabbit anti‐human Vimentin polyclonal antibody (BS91440), and rabbit anti‐human Snail polyclonal antibody (BS91262; Bioworld Technology).

### 2.4 Online database analysis

The expression profiles of LRRC59 in various cancers were analyzed by TIMER2.0 online database (http://timer.comp‐genomics.org/).^11^ Correlation analysis between LRRC59 expression and survival in BC patients was based on GEPIA2 online database (http://gepia2.cancer‐pku.cn/).^12^ The UALCAN database (http://ualcan.path.uab.edu) was used to explore the role of LRRC59 and its status of methylation in BC.^13^ Furthermore, the prognostic significance of LRRC59 methylation was assessed using the MethSurv database (http://biit.cs.ut.ee/methsurv/), which is known as an online tool for multivariable survival analysis based on DNA methylation data.^14^

### 2.5 Differentially expressed gene (DEG) analysis

BC patients in TCGA were divided into high and low LRRC59 expression groups at the median score of LRRC59 expression. DEGs between low and high expression groups were analyzed by the R package DESeq2^15^ with and adjusted *p*‐value <0.05 and |log_2_‐fold‐change (FC) set at |>1 as the threshold of DEGs. Correlations between the expression of the top 10 DEGs and LRRC59 were evaluated by Spearman's correlation analysis.

### 2.6 Gene functional enrichment analysis

Functional enrichment, Gene Ontology (GO), and Kyoto Encyclopedia of Genes and Genomes (KEGG) analyses were performed by the R package GO plot (version 1.0.2).^16^ Gene set enrichment analysis (GSEA) was performed with the R package cluster Profiler,^17^ using adjusted *p*‐value <0.05 as significant enriched function or pathway terms.

### 2.7 Immune infiltration

We used the cell type identification by estimating relative subsets of RNA transcripts (CIBERSORT) to identify and calculate 22 immune cells, consisting of NK cells, naive, and memory B cells, myeloid subsets, plasma cells, and 7T‐cell types.

### 2.8 Nomogram construction and validation

To predict the OS probability, a nomogram was constructed and validated by the calibration plot and time‐dependent ROC curve using the R package RMS (version 5.1‐4). The time‐dependent ROC curve was performed using the time ROC package.

### 2.9 Cell culture and transfection

SV‐HUC‐1 cells and T24, 5637, and J82 cells were cultured respectively in Hams F‐12K and RPMI 1640 medium containing 10% FBS and 1% penicillin streptomycin at 37°C in 5% CO_2_. Cells of logarithmic phase were digested with 0.25% trypsin to adjust the cell concentration to 1 × 10^5^/mL, and 1 mL cell suspension was inoculated per well in a 6‐well plate. After T24 and 5637 cells grew to 70%–80% of the plate area, they were transfected with shLRRC59#1, shLRRC59#2, shLRRC59#3, and shLRRC59#NC according to Lipofectamine3000 instructions.

### 2.10 Quantitative real‐time polymerase chain reaction (qRT‐PCR)

After transfection, total RNA was extracted using the TRIzol kit according to the manufacturer's instructions, and then reverse transcribed using a Bio‐Rad qRT–PCR instrument. Data calculation was performed by the 2^−ΔΔCt^ method. The primer sequences used are as follows:

LRRC59:

Forward: 5′ TGACTACTCTACCGTCGGATTT3′

Reverse: 5′ TTCAGGTCCAACCACTTCAGG 3′

GAPDH:

Forward: 5′ GGGAAACTGTGGCGTGAT3′

Reverse: 5′ GTGGTCGTTGAGGGCAAT3′

### 2.11 Immunohistochemical (IHC) analysis

After sectioning, the tissue samples were dewaxed with xylene, rehydrated with graded alcohol, and incubated with citrate antigen retrieval solution. After removing endogenous peroxidase with 3% hydrogen peroxide, samples were blocked with 5% bovine serum albumin (BSA) and then incubated with the primary antibody LRRC59 at a working concentration of 1:500 in a humid box overnight at 4°C. After washing with PBS, culture was continued by adding the secondary HRP‐labeled goat anti‐rabbit antibody was added at a working concentration of 1:200 at room temperature for 30 min. The sample was then washed with PBS, counter‐stained with DAB and hematoxylin, dried at room temperature, mounted with neutral resin mounting, and finally observed under a microscope. The staining intensity is expressed as 0, negative; 1, weakly positive; 2, moderately positive; and 3 strongly positive, and the staining area is expressed as 0 (≤9%), 1 (10%–25%), 2 (26%–50%), 3 (51%–80%), and 4 (≥81%) based on positive cells determined by two pathologists independently. The staining score = intensity score + area score, where 0–4 points are expressed as low expression, and 5–7 points as high expression.

### 2.12 Western blot assay

Cultured cells were collected from each group, and proteins were extracted using TPER lysate, boiled and deformed after measuring the concentration and subjected to SDS‐PAGE gel electrophoresis by electrotransferring them at 200 mA for 1 h to the 0.45 μm PVDF membrane, which was blocked by 5% BSA for 1 h and then incubated overnight at 4°C with primary antibodies (LRRC59 antibody 1:2000, β‐tubulin antibody 1:2000, β‐actin antibody 1:2000, E‐cadherin antibody 1:1000, vimentin antibody 1:1000, Snail antibody 1:500). After TBST washing, HRP‐labeled secondary antibodies (goat anti‐rabbit antibody 1:2000, goat anti‐mouse antibody 1:2000) were added and incubated at room temperature for 1 h. After TBST washing, ECL chemiluminescence reagent was developed and photographed by LI‐COR exposed to analyze the gray level of western blots.

### 2.13 CCK‐8 assay

T24 and 5637 cells in shLRRC59#1 and shLRRC59#NC groups were digested with trypsin, centrifuged, resuspended in culture medium, adjusted to a cell concentration of 104/mL, seeded in 96‐well plates with three duplicate each well, inoculated in 200 μL in each well, transferred into 100 μL culture medium containing 10 μL CCK‐8 after cell attachment, and incubated for 2 h. At 0‐, 24‐, 48‐, 72‐ and 96‐h inoculation, absorbance was measured at 450 nm with a microplate reader.

### 2.14 Colony formation assay

After transfection of T24 and 5637 cells with LRRC59 shRNA plasmid for 48 h, cells were digested with trypsin and centrifuged. Cells were seeded into 6‐well plates at a density of 600 cells/well. After routine culture of the cells for 7 days, crystal violet solution staining was performed to observe colony formation.

### 2.15 Wound‐healing assay

Transfected T24 and 5637 cells were inoculated into 6‐well plates in triplicate, and cultured until approximately 90% confluence. Using a 200 μL pipette tip, a standardized scratch was made. After washing off the cell debris with PBS and replacing the serum‐free medium, cells were photographed at 0 and 24 h, and cell mobility was calculated using the following equation: Cell mobility = (initial scratch area–endpoint time area) ÷ initial scratch area.

### 2.16 Transwell assay

Transwell chambers were placed into 24‐well plates. Complete medium (500 μL) was added to the lower chamber. After adjusting the cell concentration to 2.5 × 105 cells/mL with serum‐free medium, 200 μL cells were added to the upper chamber of the insert, with three duplicate wells in each group. After 48‐h culture and discarding the solution in the upper chamber, cells were wiped off with a cotton swab, stained with 1% crystal violet for 20 min, observed under a 200× microscope and countered in three randomly selected fields to evaluate the cell migration ability. Cell invasion assay was performed by coating the transwell chambers with matrigel, and the remaining steps were the same as before to determine cell invasion.

### 2.17 Statistical analysis

SPSS 25.0, GraphPad 8.0 software, and R software V3.6.3 were applied for statistical analysis. All experiments were performed in triplicate. Data are expressed as mean ± standard deviation (SD). Comparison between two groups was performed by independent sample *t*‐test and comparison between multiple groups was performed by multiple *t*‐test. Survival analysis was performed by the Kaplan–Meier method, with the cutoff values set at the median expression level of LRRC59. Patient survival prognosis was analyzed by univariate and multivariate cox regression analyses. *p*‐ values <0.05 were considered statistically significant.

## 3 RESULTS

### 3.1 LRRC59 expression in BC

**FIGURE 1:**
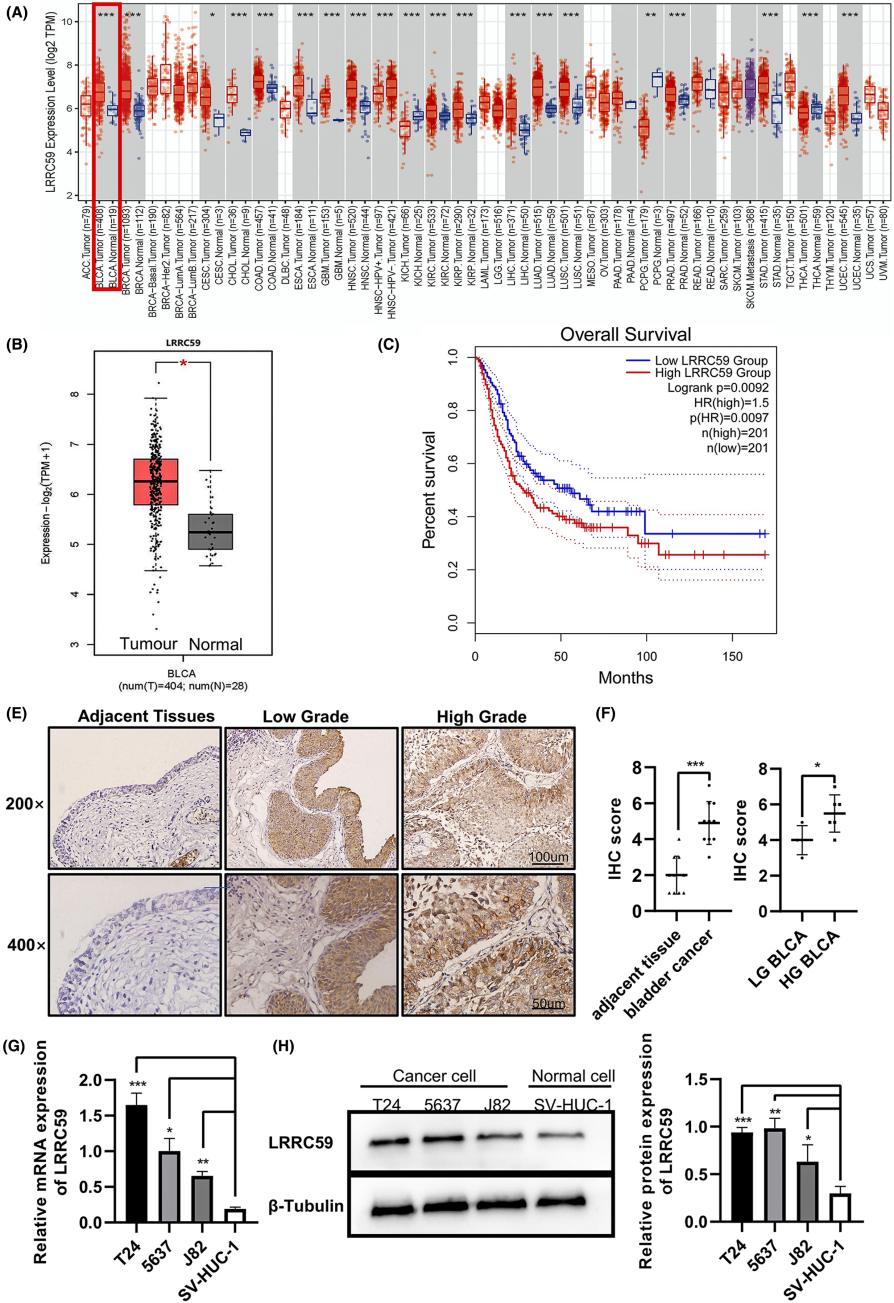
LRRC59 is highly expressed in bladder cancer (BC) patients and BC cell lines. (A) TIMER database analysis of LRRC59 expression in pan‐cancer. (B) GEPIA database analysis of LRRC59 expression in BC, where the red box indicates the tumor samples (*n* = 404) and the gray one represents the normal tissues (*n* = 28). (C) Expression levels of LRRC59 in BC and matched normal tissues in TCGA database. (D) Receiver operating characteristic (ROC) curves for classifying BC versus normal bladder tissues in the TCGA database. (E, F) Correlation of LRRC59 expression with CTLA4 and PDCD1 expression in BC in the TCGA database. (G, H) LRRC59 expression in BC and adjacent tissues was assessed by immunohistochemical staining. LRRC59 expression in three BC cell lines (T24, 5637 and J82) versus normal bladder cell line SV‐HUC‐1 was detected by qRT‐PCR (I) and western blot (J). *p < 0.05; **p < 0.01; ***p < 0.001.

Differential expression of LRRC59 in pan‐cancers was analyzed based on TIMER database, and the result showed that LRRC59 expression was elevated in bladder, breast, colon cancers, and cholangiocarcinoma (Figure 1A). The GEPIA2 online tool was used to further calculate feature scores by the mean of log_2_ (TPM + 1) of LRRC59 in BC and normal bladder tissues by setting the |Log_2_FC| cutoff as 1 for expression and the *p*‐value cutoff as 0.05. The result showed that the RNA expression of LRRC59 in BC tissue was significantly higher than that in normal bladder tissue (*p* < 0.001; Figure 1B). In addition, LRRC59 was highly expressed in 19 paired BC tissues (*p* < 0.001; Figure 1C). Furthermore, the ROC curve indicated that LRRC59 expression had good predictive power with an area under the curve (AUC) of 0.808 (95% confidence interval [CI] = 0.737–0.879) and can be used to discriminate BC tissue from normal tissue (Figure 1D). In addition, we verified the correlation between LRRC59 and immune checkpoint‐associated gene, and found that LRRC59 was significantly correlated with CTLA4 and PDCD1 (*p* < 0.001; Figure 1E,F).

Using immunohistochemistry staining to detect the clinical samples, those with cell expression of LRRC59 were stained brownish‐yellow mainly in the cytoplasm and with a small amount in the nucleus. LRRC59 expression in BC tissue was significantly higher than that in adjacent noncancerous tissue (*p* < 0.001; Figure 1G). Also, its expression in high‐grade BC tissue was significantly higher than that in low‐grade BC tissue (*p* < 0.05; Figure 1H). The expressions of LRRC59 in the normal urothelial cell line SV‐HUC‐1 and BC cell lines T24, 5637, and J82 were detected by qRT‐PCR and western blot. The results showed that the expression of LRRC59 in T24, 5637, and J82 cells was significantly higher than that in SV‐HUC‐1 cells (*p* < 0.05; Figure 1I,J), with the highest expression in T24 cells, followed by 5637 cells (*p* < 0.05; Figure 1I,J). Therefore, we selected T24 cells and 5637 cells for subsequent experiments.

### 3.2 Correlation between LRRC59 expression and clinicopathologic features

**FIGURE 2:**
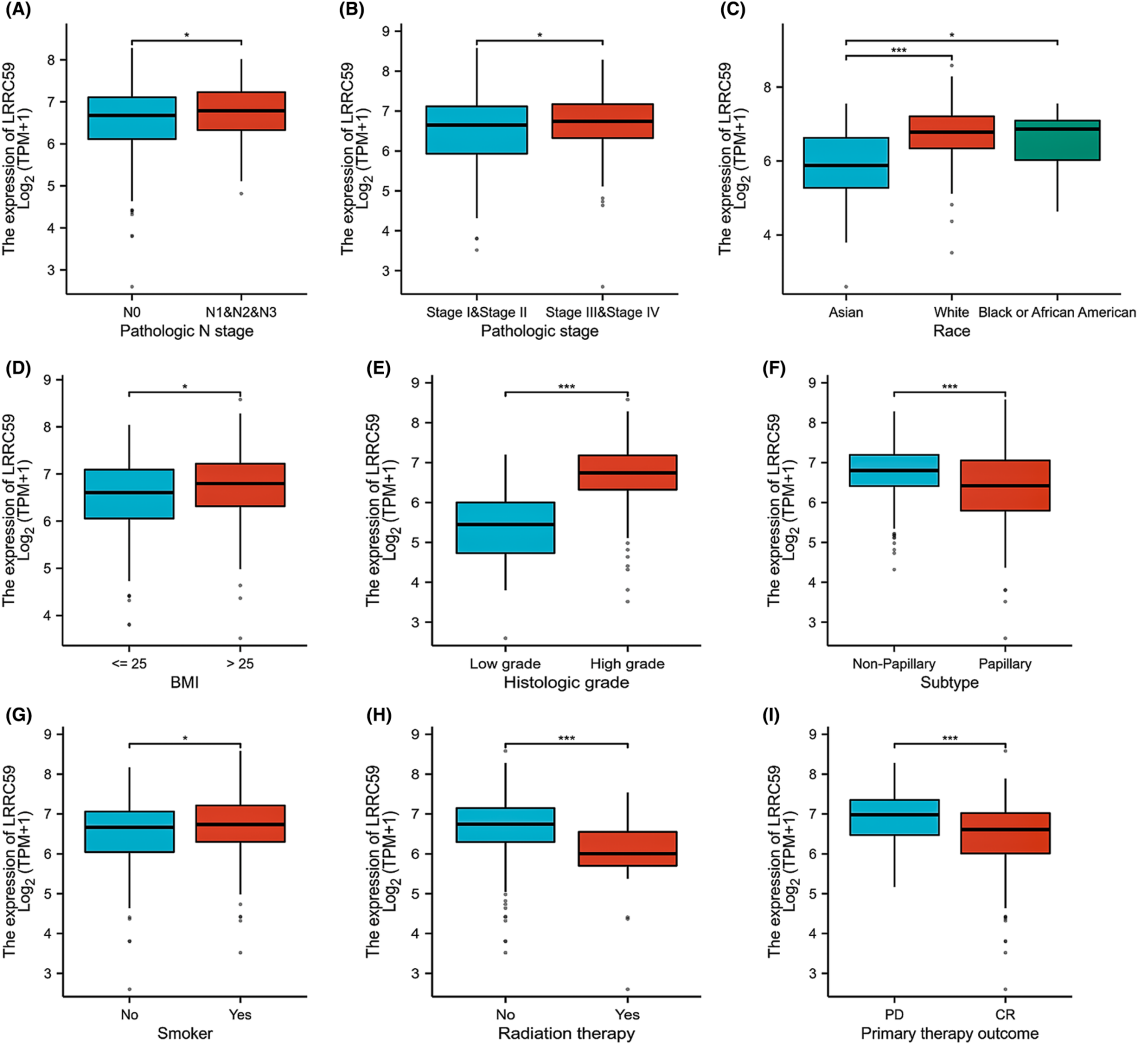
Correlation between LRRC59 expression and clinicopathologic characteristics. Boxplots are shown for (A) N stage; (B) pathologic stage; (C) race; (D) BMI; (E) histologic grade; (F) subtype; (G) smoking; (H) radiation therapy; (I) primary therapy outcome. *p < 0.05; ***p < 0.001.

LRRC59 overexpression was significantly correlated with the pathologic stage (stage III and IV; stage I and, *p* < 0.05), N stage (N1&2&3 vs. N0, *p* < 0.05), histologic grade (high grade vs. low grade, *p* < 0.001), OS (*p* < 0.01), disease‐specific survival (DSS; *p* < 0.05), and progress‐free interval (PFI; *p* < 0.05; Table 1; Figure 2). The results of the univariate analysis showed that there were certain clinicopathologic differences between LRRC59 high and low expression groups in terms of the histologic grade (odds ratio [OR] = 0.046, 95% CI = 0.003–0.333, *p* = 0.003), subtype (OR = 0.508, 95% CI = 0.331–0.774, *p* = 0.002), race (OR = 2.373, 95% CI = 1.377–4.195, *p* = 0.002), and primary therapy outcome (OR = 0.516, 95% CI = 0.319–0.828, *p* = 0.003; Table 2).

**TABLE 1:** Clinicopathologic features of high and low LRRC59 expression groups.

Characteristics	Total (N)	Odds ratio(OR)	*p*‐value
T stage (T3&T4 vs. T1&T2)	374	1.278 (0.829–1.976)	0.268
N stage (N1&N2&N3 vs. N0)	366	1.370 (0.892–2.110)	0.152
M stage (M1 vs. M0)	207	1.023 (0.286–3.504)	0.971
Pathologic stage (Stage III&Stage IV vs. Stage I&Stage II)	406	1.310 (0.864–1.991)	0.204
Age (>70 vs. ≤70)	408	0.961 (0.649–1.421)	0.842
Race (White vs. Asian&Black or African American)	391	2.373 (1.377–4.195)	0.002
Gender (Male vs. Female)	408	0.927 (0.595–1.441)	0.736
Histologic grade (Low grade vs. High grade)	405	0.046 (0.003–0.222)	0.003
Radiation therapy (Yes vs. No)	382	0.217 (0.061–0.598)	0.007
Subtype (Papillary vs. Non‐papillary)	403	0.508 (0.331–0.774)	0.002
Lymphovascular invasion (Yes vs. No)	281	0.888 (0.554–1.421)	0.620
Smoker (Yes vs. No)	395	1.277 (0.821–1.994)	0.279
Primary therapy outcome (PR&CR vs. PD&SD)	351	0.516 (0.319–0.828)	0.007
Weight (>80 vs. ≤80)	365	0.958 (0.633–1.447)	0.837
Height (>170 vs. ≤170)	359	1.148 (0.756–1.746)	0.517
BMI (>25 vs. ≤25)	358	1.652 (1.082–2.532)	0.020

**TABLE 2:** Correlation of LRRC59 expression with clinicopathologic features in 408 patients with bladder cancer (BC).

Features	Low expression of LRRC59	High expression of LRRC59	*p*
*N*	204	204	
T stage, *n* (%)			0.681
T1	2 (0.5%)	1 (0.3%)	
T2	65 (17.4%)	54 (14.4%)	
T3	94 (25.1%)	100 (26.7%)	
T4	29 (7.8%)	29 (7.8%)	
N stage, *n* (%)			0.471
N0	127 (34.7%)	110 (30.1%)	
N1	20 (5.5%)	26 (7.1%)	
N2	36 (9.8%)	39 (10.7%)	
N3	3 (0.8%)	5 (1.4%)	
M stage, *n* (%)			1.000
M0	108 (52.2%)	88 (42.5%)	
M1	6 (2.9%)	5 (2.4%)	
Pathologic stage, *n* (%)			0.512
Stage I	1 (0.2%)	1 (0.2%)	
Stage II	71 (17.5%)	59 (14.5%)	
Stage III	70 (17.2%)	70 (17.2%)	
Stage IV	61 (15%)	73 (18%)	
Radiation therapy, *n* (%)			0.007
No	173 (45.3%)	188 (49.2%)	
Yes	17 (4.5%)	4 (1%)	
Primary therapy outcome, *n* (%)			0.011
PD	27 (7.7%)	41 (11.7%)	
SD	11 (3.1%)	18 (5.1%)	
PR	8 (2.3%)	14 (4%)	
CR	133 (37.9%)	99 (28.2%)	
Gender, *n* (%)			0.822
Female	52 (12.7%)	55 (13.5%)	
Male	152 (37.3%)	149 (36.5%)	
Race, *n* (%)			<0.001
Asian	35 (9%)	9 (2.3%)	
Black or African American	10 (2.6%)	13 (3.3%)	
White	150 (38.4%)	174 (44.5%)	
Age, *n* (%)			0.920
≤70	114 (27.9%)	116 (28.4%)	
>70	90 (22.1%)	88 (21.6%)	
Weight, *n* (%)			0.920
≤80	102 (27.9%)	99 (27.1%)	
>80	85 (23.3%)	79 (21.6%)	
Height, *n* (%)			0.588
≤170	83 (23.1%)	73 (20.3%)	
>170	101 (28.1%)	102 (28.4%)	
BMI, *n* (%)			0.027
≤25	87 (24.3%)	62 (17.3%)	
>25	96 (26.8%)	113 (31.6%)	
Histologic grade, *n* (%)			< 0.001
High grade	183 (45.2%)	201 (49.6%)	
Low grade	20 (4.9%)	1 (0.2%)	
Subtype, *n* (%)			0.002
Non‐papillary	121 (30%)	150 (37.2%)	
Papillary	81 (20.1%)	51 (12.7%)	
Smoker, *n* (%)			0.332
No	60 (15.2%)	49 (12.4%)	
Yes	140 (35.4%)	146 (37%)	
OS event, *n* (%)			0.003
Alive	130 (31.9%)	99 (24.3%)	
Dead	74 (18.1%)	105 (25.7%)	
DSS event, *n* (%)			0.026
Alive	146 (37.1%)	126 (32%)	
Dead	50 (12.7%)	72 (18.3%)	
PFI event, *n* (%)			0.012
Alive	130 (31.9%)	104 (25.5%)	
Dead	74 (18.1%)	100 (24.5%)	
Age, median (IQR)	69 (60, 76)	68 (61, 76)	0.627

### 3.3 Identification of DEGs in BC

**FIGURE 3:**
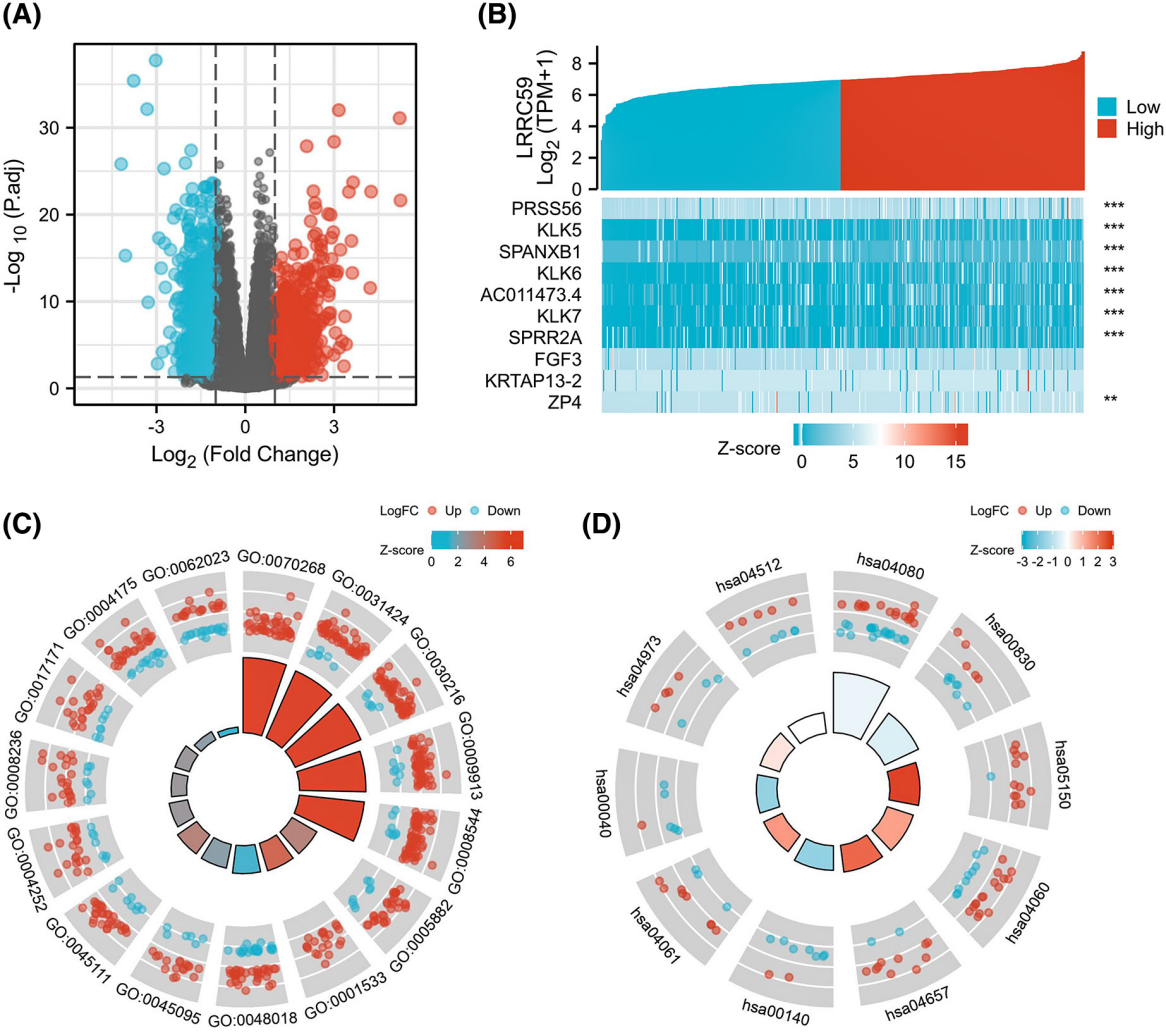
LRRC59‐related differentially expressed genes (DEGs) and functional enrichment analysis of LRRC59 in bladder cancer using GO and KEGG. (A) Volcano plot of DEGs. Blue dots indicate the downregulated DEGs and red dots indicate upregulated DEGs. (B) Heatmap presentation of top 10 DEGs. (C) GO analysis of DEGs. (D) KEGG pathway analysis of DEGs.

A total of 2899 genes were found to be differentially expressed in LRRC59 high and low expression groups, of which 723 were upregulated and 2176 were downregulated (adjusted *p*‐value <0.05, |Log_2_‐FC| > 1; Figure 3A). The relationship between the top 10 DEGs (including PRSS56, KLK5, SPANXB1, KLK6, AC011473.4, KLK7, SPRR2A, FGF3, KRTAP13‐2, and ZP4) is presented in a heatmap (Figure 3B; Table S1).

### 3.4 GO, KEGG, and GSEA functional enrichment analyses

**FIGURE 4:**
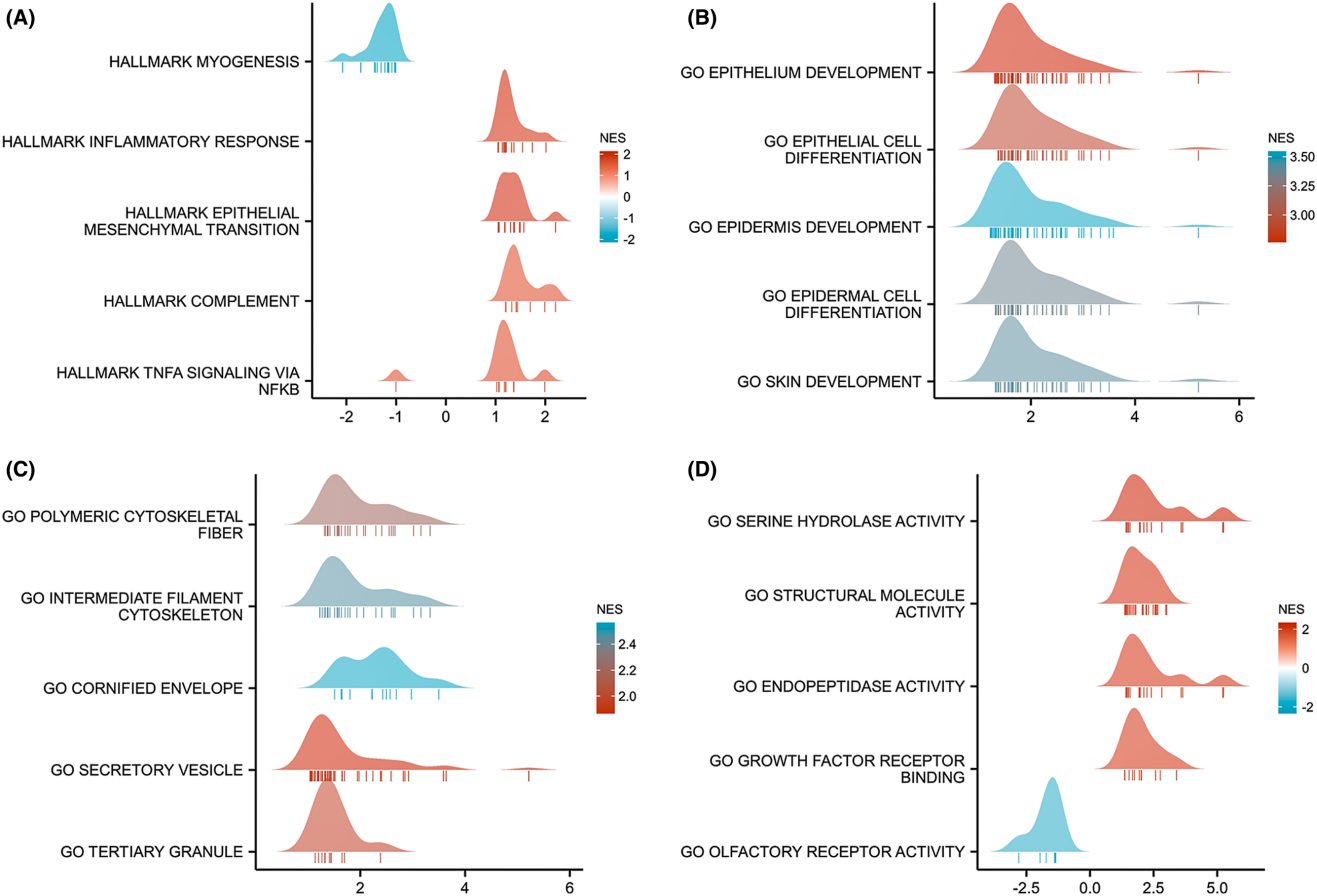
Gene set enrichment analysis (GSEA) of DEGs. (A) GSEA analysis of the hallmark gene sets. (B) GSEA analysis of the biological processes (BPs) of Gene Ontology (GO) gene sets. (C) GSEA analysis of the cellular component (CC) of GO gene sets. (D) GSEA analysis of the molecular function (MF) of GO gene sets.

GO enrichment analysis including the biological process (BP), cellular composition (CC), and molecular function (MF) revealed that these DEGs were enriched in different GO terms such as epidermis development, epidermal cell differentiation, collagen‐containing extracellular matrix, endopeptidase activity, and receptor ligand activity (Figure 3C; Table S2). Additionally, the result of KEGG pathway analysis demonstrated that significant DEG‐enriched pathways included neuroactive ligand–receptor interaction, retinol metabolism, *Staphylococcus aureus* infection, cytokine–cytokine receptor interaction, and IL‐17 signaling pathway (Figure 3D; Table S3). Next, GSEA showed that epithelial–mesenchymal transition (EMT) and epithelial cell differentiation BPs were more significantly enriched in LRRC59 high expression group, suggesting that the high expression of LRRC59 conferred a cell proliferation and metastasis phenotype in BC (Figure 4A–D).

### 3.5 Correlation between methylation and LRRC59 expression

**FIGURE 5:**
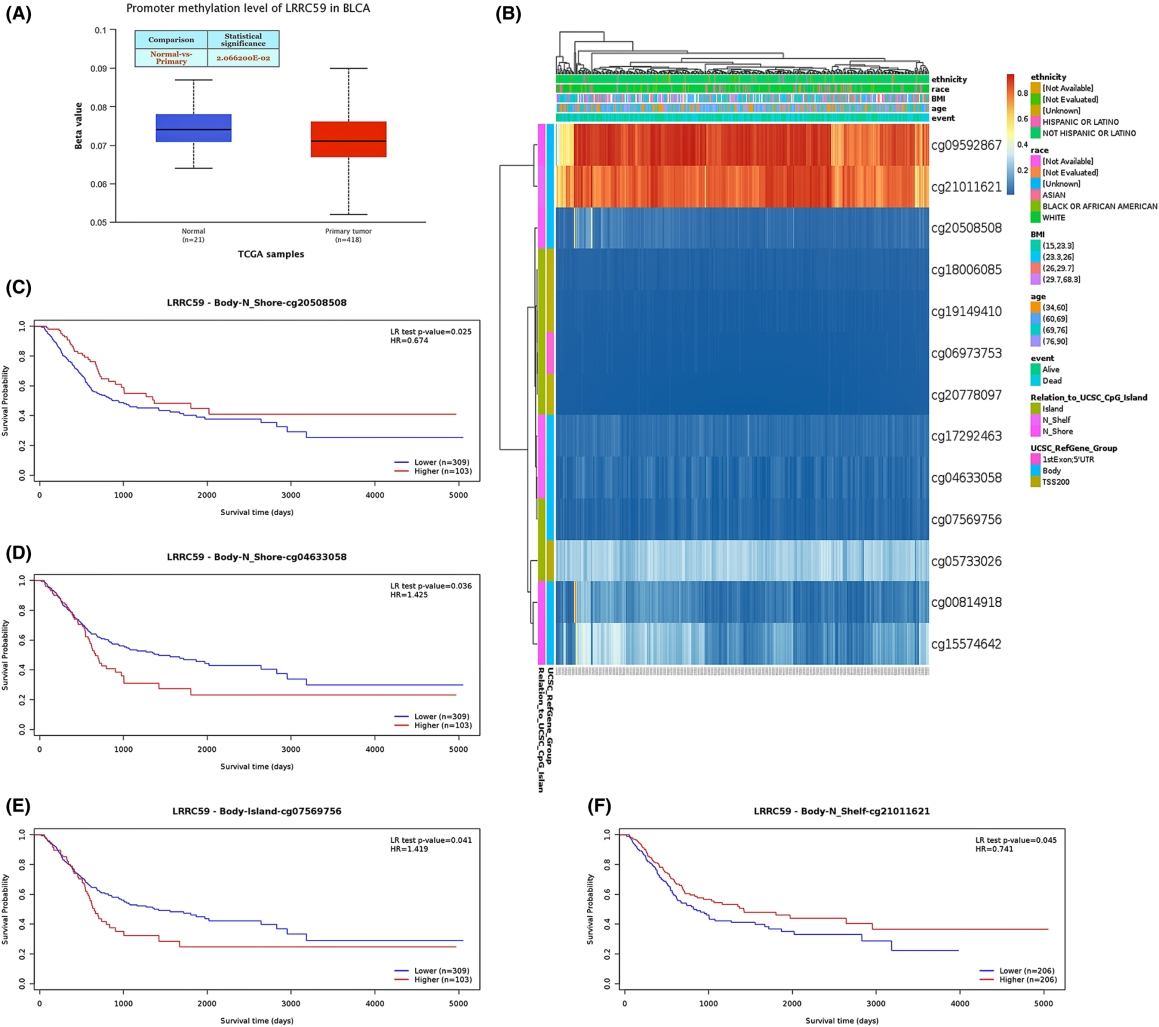
DNA methylation level of LRRC59 and its function on prognosis of patients with bladder cancer (BC). (A) The promoter methylation level of LRRC59 in BC was obtained from the UALCAN database. (B) Correlation between LRRC59 mRNA expression level and methylation level. (C–F) Kaplan–Meier survival curves for several methylation sites of LRRC59.

The correlation between the LRRC59 expression level and the methylation status was analyzed using online tools. It was found that the DNA methylation level at the promoter in BC primary tumor tissues was significantly lower than that in the normal bladder tissues based on the UALCAN database (*p* < 0.05; Figure 5A). In addition, most methylation sites were hypomethylated in the DNA sequences of LRRC59 in BC, and the degree of methylation was correlated with the patient survival outcome (Figure 5B). Finally, several methylation sites indicated a poor prognosis, including cg20508508 and cg21011621 (Figure 5C–F).

### 3.6 Correlation between LRRC59 expression and immune infiltration

**FIGURE 6:**
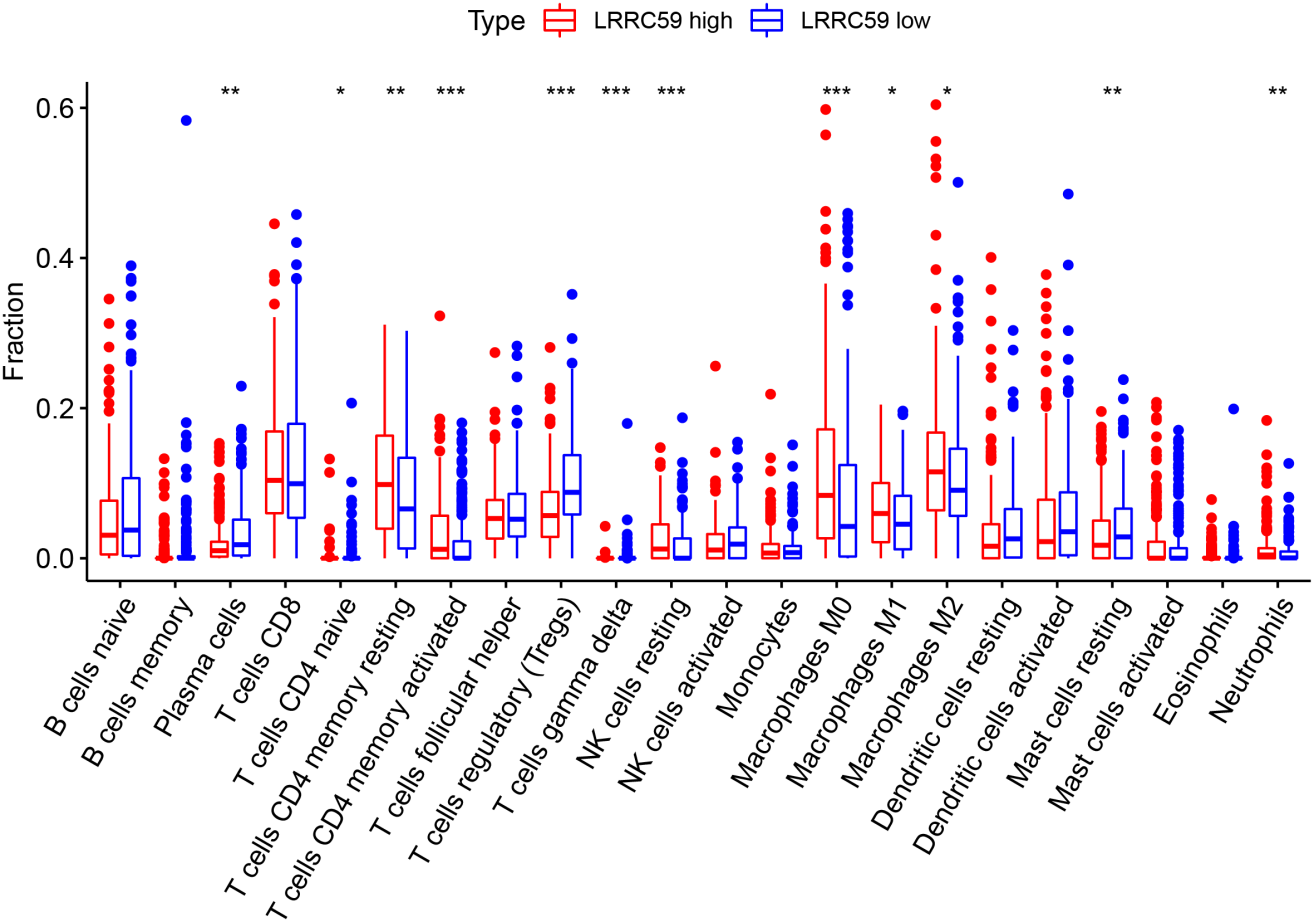
The distinctions of the immune cells distribution between high LRRC59 (red) and low LRRC59 (blue) expression in BC were shown in box plot.

To explore the immune infiltration in BC, the CIBERSORT methodology and R software were used to obtain the distribution of distinction of immune cells between high LRRC59 expression and low LRRC59 expression in BC. Contrasted with low LRRC59 expression BC, the infiltration abundance of resting memory CD4 T cells, memory activated CD4 T cells, resting NK cells, macrophages M0, M1, M2, and neutrophils increased in high LRRC59 expression BC, while the infiltration abundance of plasma cells, naïve CD4 T cells and regulatory T cells (Tregs) declined in high LRRC59 expression BC (Figure 6).

### 3.7 Prognostic significance of LRRC59 in BC

**FIGURE 7:**
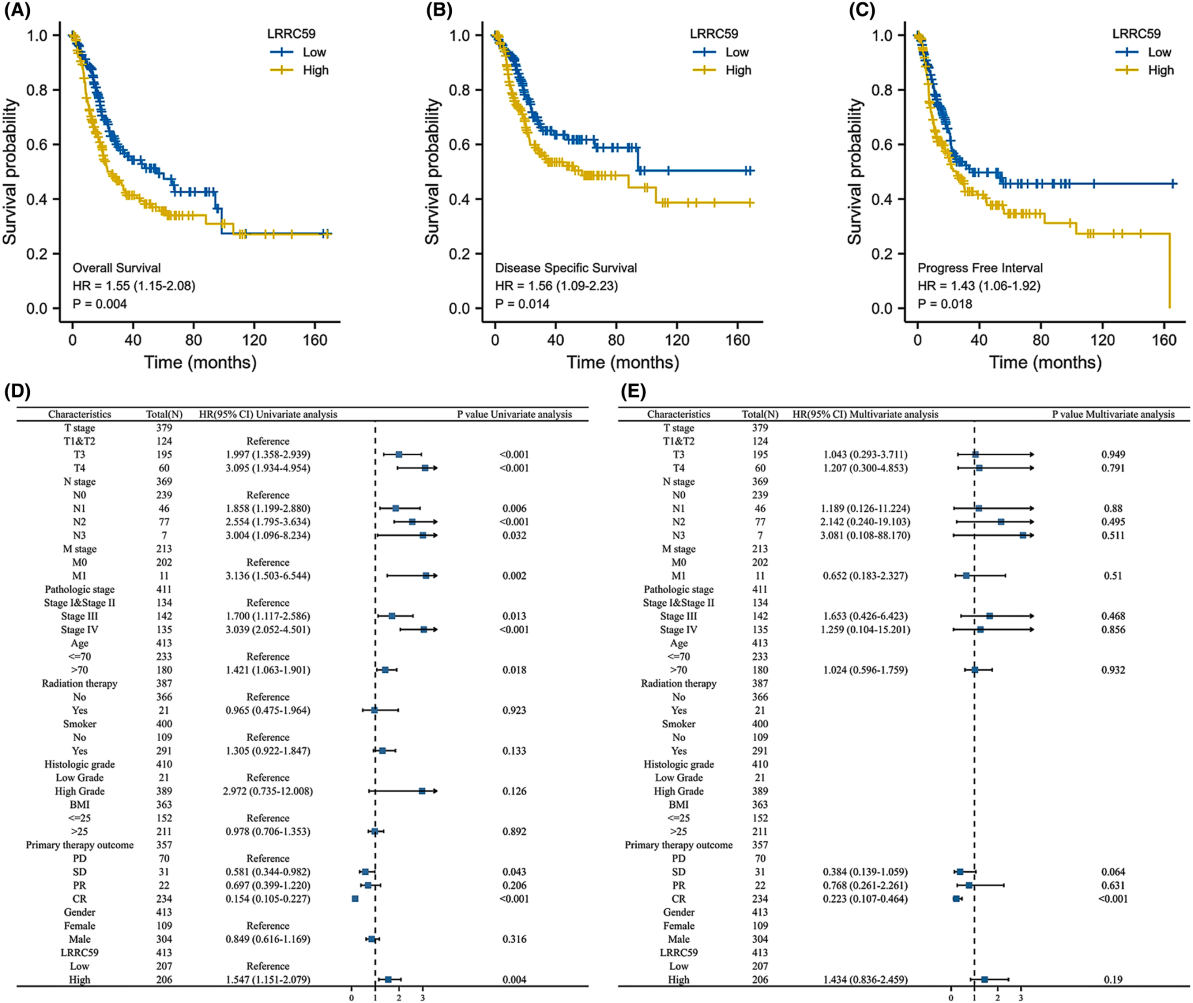
Prognostic values of LRRC59 expression in patients with bladder cancer (BC) evaluated by the Kaplan–Meier method. Overall survival (A), disease‐specific survival (B), and progress‐free interval (C) for BC patients with high versus low LRRC59. Univariate (D) and multivariate (E) Cox analysis for overall survival.

The correlation between LRRC59 expression and the survival prognosis of BC patients was analyzed by the Kaplan–Meier method. The median level of LRRC59 expression was used as a cutoff score, and the patients were divided into LRRC59 high and low expression groups. OS, DSS, and PFI in LRRC59 high expression group were worse than those in LRRC59 low expression group (OS: hazard ratio [HR] = 1.55, 95% CI = 1.15–2.08, *p* < 0.01; DSS: HR = 1.56, 95% CI = 1.09–2.23, *p* < 0.05; PFI: HR = 1.43, 95% CI = 1.06–1.92, *p* < 0.05; Figure 7A).

Univariate and multivariate Cox regression analyses were conducted to identify prognostic indicators. The results of univariate analysis showed that LRRC59 expression (HR = 1.547, 95% CI = 1.151–2.079, *p* < 0.01), T stage (T3 HR = 1.997, 95% CI = 1.358–2.939, *p* < 0.001; T4 HR = 3.095, 95% CI = 1.934–4.954, *p* < 0.001), M stage (HR = 3.136, 95% CI = 1.503–6.544, *p* < 0.01), pathologic stage (stage III HR = 1.700, 95% CI = 1.117–2.586, *p* < 0.05; stage IV HR = 3.039, 95% CI = 2.052–4.501, *p* < 0.001), and age (HR = 1.421, 95% CI = 1.063–1.901, *p* < 0.05) were independent factors of OS in BC patients (Figure 7B,C).

**FIGURE 8:**
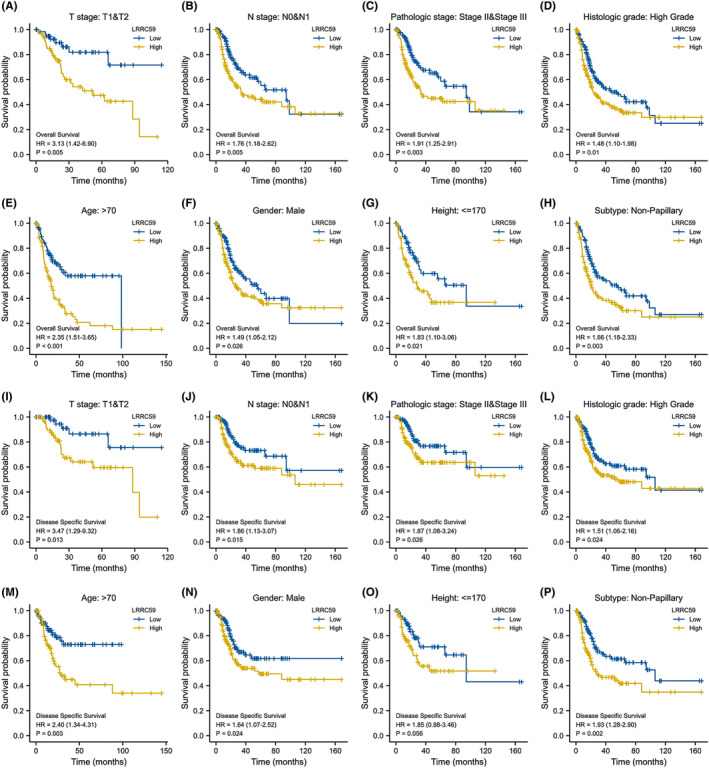
Prognostic values of LRRC59 expression in patients with bladder cancer (BC) evaluated by the Kaplan–Meier method in different subgroups. (A–H) Overall survival curves of T1 and T2, N0 and N1, stage II and III, high grade, age>70 years, male, height ≤ 170, and non‐papillary subgroups between BC patients with high or low LRRC59 (I–P) D‐specific survival curves of T1 and T2, N0 and N1, stage II and III, high grade, age > 70 years, male, height ≤ 170, and non‐papillary subgroups between BC patients with high or low LRRC59.

Regardless of OS or DSS, the prognosis of patients with high expression of LRRC59 was more unfavorable in several subgroups, including T1 and T2, N0 and N1, M0, stage II and III, high grade, age > 70 years, male, height ≤ 170, and non‐papillary subgroups (all *p* < 0.05; Figure 8A–P).

### 3.8 Establishment of the nomogram

**FIGURE 9:**
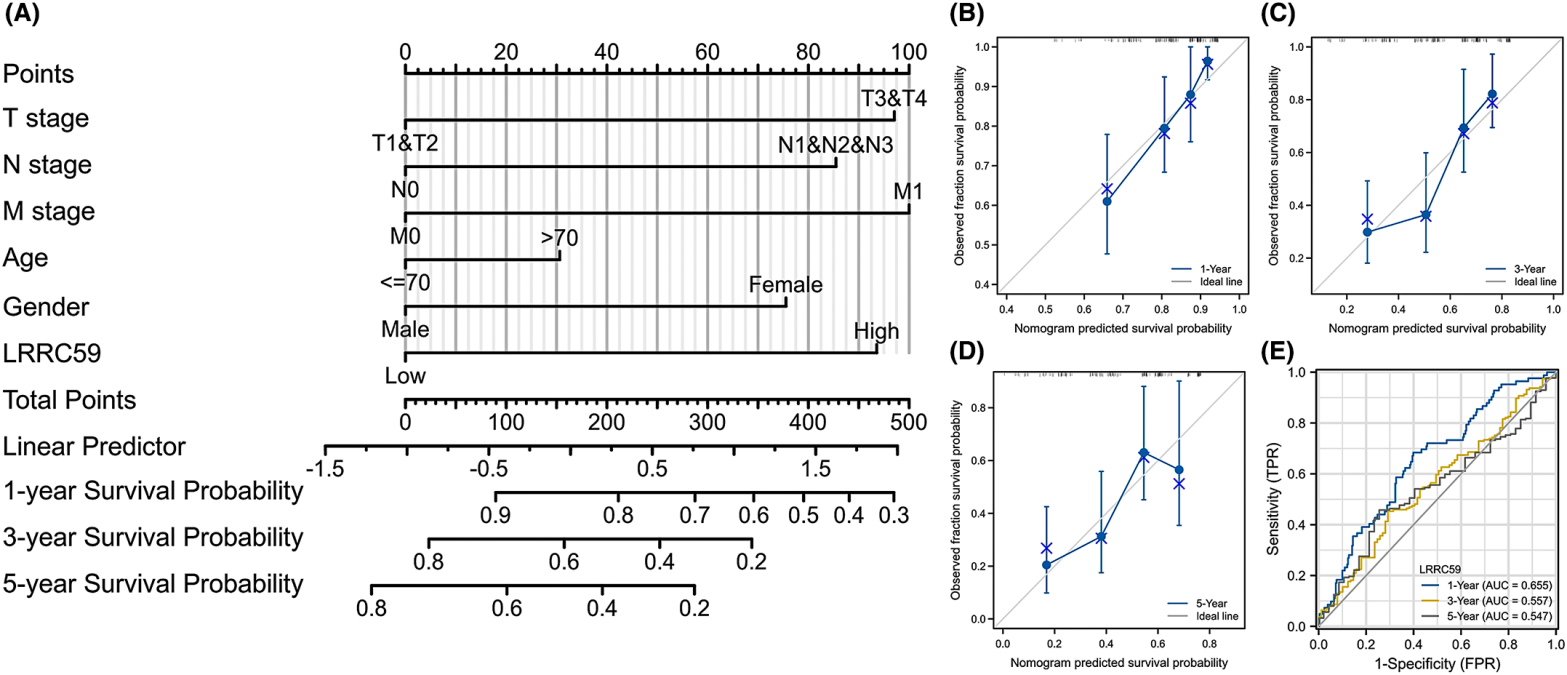
Nomogram for prediction of 1‐, 3‐, and 5‐year overall survival (OS) of patients with bladder cancer (BC). (A) Nomogram for predicting 1‐, 3‐, and 5‐year OS of BC patients. (B–D) The calibration curves validate the consistency between the actual outcome and the predicted result for 1‐, 3‐, and 5‐year OS. (E) Time‐dependent ROC curves based on LRRC59 expression for 1‐, 3‐, and 5‐year OS probability.

To predict the prognosis of BC patients, a nomogram based on the independent factors of OS was generated, in which a higher total number of points means a worse prognosis (Figure 9A). In addition, the prediction efficacy of the nomogram was assessed by calibration curves (Figure 9B–D), and the result showed that the 1‐, 3‐ and 5‐year AUC values were 0.655, 0.557, and 0.547, respectively (Figure 9E), indicating that the nomogram was appropriate.

### 3.9 LRRC59 knockdown inhibits BC cell proliferation

**FIGURE 10:**
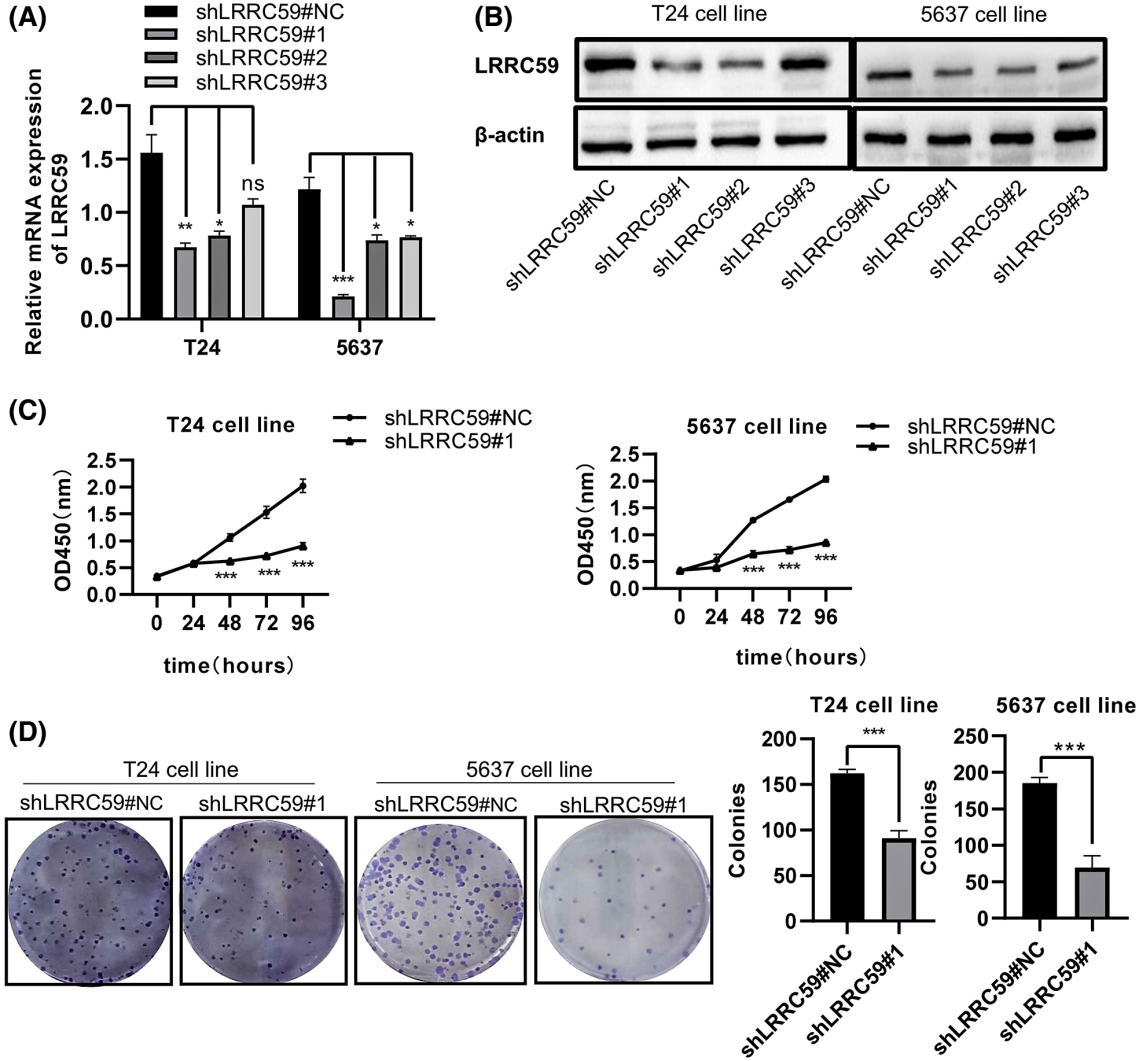
Decreased expression of LRRC59 inhibits the proliferation of bladder cancer (BC) cell lines. LRRC59 expression in T24 and 5637 BC cells transfected with shLRRC59#1, 2, 3 or its associated negative control (shLRRC59#NC) was detected by qRT‐PCR (A) and western blot (B). Cell proliferation level was analyzed in T24 and 5637 BC cells transfected with shLRRC59 or control by cell‐counting kit‐8 assay (C) and colony‐forming assay (D). *p < 0.05; **p < 0.01; ***p < 0.001; ns means no significant.

To explore the role of LRRC59 in BC, LRRC59 expression was knocked down by shRNA in BC cells. The results of qRT‐PCR and western blot assays showed that shRNA1 and shRNA2 significantly reduced the LRRC59 expression at the RNA and protein levels in T24 and 5637 cells with the most significant decrease in shLRRC59#1 group (*p* < 0.05; Figure 10A,B), and then shLRRC59#1 was selected for subsequent experiments. After knocking down LRRC59 expression in BC cells, CCK8 assay showed that compared with the control group, the proliferation ability of T24 and 5637 cells was significantly decreased (*p* < 0.001; Figure 10C). The colony formation assay also showed that the colony size and the number of T24 and 5637 cells were significantly inhibited compared with the control group (*p* < 0.001; Figure 10D).

### 3.10 LRRC59 knockdown inhibits BC cell migration and invasion

**FIGURE 11:**
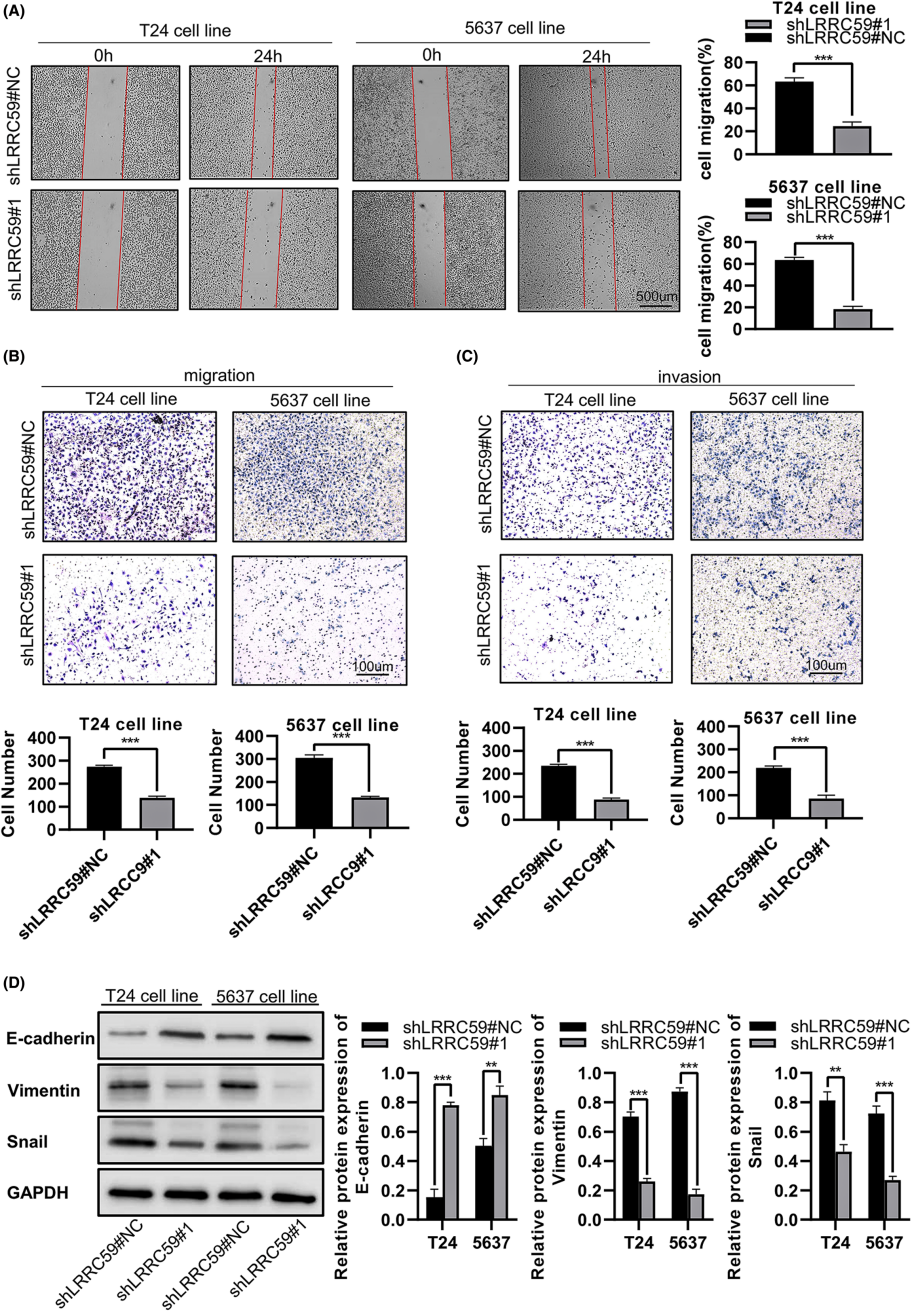
Decreased expression of LRRC59 inhibits bladder cancer (BC) cell migration. Cell migration levels were analyzed in T24 and 5637BC cells transfected with shLRRC59 or negative control by wound‐healing assay. (A) and transwell assay (B). (C) Cell invasion ability was analyzed in T24 and 5637 BC cells transfected with shLRRC59 or negative control by transwell assay. (D) The protein expression level of epithelial–mesenchymal transition‐associated markers was measured by western blotting in T24 and 5637 BC cells transfected with shLRRC59 or negative control. *p < 0.05; **p < 0.01; ***p < 0.001.

After knocking down LRRC59 expression in T24 and 5637 cells, wound‐healing assay showed that their migration rate was significantly lower than that in the control group (T24 shLRRC59#NC = 69.37 ± 3.47%, T24 shRNA1 = 24.63 ± 3.64%, 5637 shLRRC59#NC = 69.37 ± 3.47%, 5637 shLRRC59#1 = 18.37 ± 2.75%, *p* < 0.001; Figure 11A). The result of transwell assay on cell migration and invasion showed that reduced LRRC59 expression significantly decreased cell migration (T24 shLRRC59#NC = 274 ± 7, T24 shLRRC59#1 = 139 ± 7, 5637 shLRRC59#NC = 305 ± 13, 5637 shLRRC59#1 = 133 ± 5, *p* < 0.001; Figure 11B), and cell invasion (T24 shLRRC59#NC = 235 ± 7, T24 shLRRC59#1 = 83 ± 4, 5637 shLRRC59#NC = 219 ± 8, 5637 shLRRC59#1 = 76 ± 2, *p* < 0.001; Figure 11C).

The above findings indicate that LRRC59 played a vital role in cancer cell migration and invasion. And the progression of this phenotype is associated with the activation of EMT.^18^, ^19^, ^20^ We therefore detected the expression of EMT‐related proteins in T24 and 5637 cells by western blotting, finding that knockdown of LRRC59 expression upregulated E‐cadherin expression and downregulated vimentin and Snail expression in T24 and 5637 cells (*p* < 0.01; Figure 11D). These results suggest that LRRC59 may affect BC cell migration and invasion by regulating EMT.

## 4 DISCUSSION

Bladder urothelial carcinoma is the most common malignancy of the urinary system characterized by high invasiveness and high recurrence rates. The incidence of BC is on the rise in recent years.^21^ Based on the classical treatment modalities such as transurethral tumor resection, intravesical chemotherapy, and radical cystectomy, drugs such as ICIs (PD‐1/PD‐L1 monoclonal antibodies) have been developed in recent years.^22^, ^23^ They have improved the outcomes of BC treatment remarkably but not fundamentally, mainly due to little knowledge about its pathogenesis, making early diagnosis and treatment difficult.^24^, ^25^ Therefore, it is necessary to find new molecular targets for the early diagnosis and treatment of BC. LRRC59 is a transmembrane protein that is mainly distributed in the ER and nuclear membrane, with its C‐terminal short region facing the ER lumen and four LRRs and coiling domains facing the cytoplasm.^4^, ^5^ LRRC59 gene or protein overexpression has been reported in several tumors.^7^, ^9^, ^26^ Recent studies have demonstrated that LRRC59 is associated with TNM stage, LNM, histological differentiation, and poor prognosis of lung cancer.^9^ Another study found that LRRC59 overexpression promoted urothelial carcinoma cell proliferation and invasion through ER stress.^27^ Additionally, LRRC59 can regulate the transport of nucleic acid‐sensitive toll‐like receptor from the ER to endosomes, playing an important role in immune responses.^28^ This article aimed to explore the role of LRRC59 in BC, and its significance as a molecular marker for the treatment and prognosis of BC.

Recently, LRRC59 has received increasing attention as a potential oncogene, and some studies have implicated LRRC59 in CIP2A nuclear import.^6^ CIP2A has been found to be highly expressed in BC, nasopharyngeal carcinoma, cholangiocarcinoma, and non‐small cell lung cancer.^29^ Further studies have demonstrated that CIP2A plays an important role in the initiation and progression of BC through regulating the EMT expression.^30^ It was found in our study that LRRC59 downregulation decreased BC cell migration and invasion. In addition, E cadherin expression was upregulated, but vimentin and Snail expression was downregulated in BC cells. The result of our enrichment analysis showed that LRRC59 affected the migration and invasion ability of BC cells by regulating their EMT process. In this study, we found that LRRC59 expression was upregulated in clinical BC specimens. The prediction results of biological information showed that OS of the patients with high LRRC59 expression was poorer, suggesting that LRRC59 plays a tumor‐promoting role in BC progression.

In recent years, immunotherapy for BC has become a hot topic.^31^, ^32^ In this study, we found that LRRC59 was significantly correlated with CTLA4 and PDCD1 in BC based on TCGA database, which are key immune checkpoint molecules. Next our immune infiltration found that LRRC59 correlated with multiple tumor‐infiltrating immune cells, such as naive CD4 T cells, gamma delta T cells, NK cells, and Tregs. It has been documented that CD4+ T cells can mediate the key to anti‐tumor by secreting cytokines.^33^ Therefore, we suggest that LRRC59 may play a key role in the immune microenvironment of BC, our findings may provide novel insight into immunotherapy targets in BC.

Our experimental results showed that knockdown of LRRC59 expression significantly inhibited BC cell proliferation and increased apoptosis of BC cells, which was consistent with the previous findings.^27^ Previous studies have suggested that LRRC59 was thought of as an intracellular binding molecule for fibroblast growth factor 1 (FGF1), and is a structure necessary for FGF1 to enter the nucleus.^34^ At present, FGF1 function has been well studied and was found to be widely involved in cell proliferation, survival, migration, and invasion.^35^ FGF1 has been shown to inhibit p53‐dependent apoptosis and arrest cell growth.^36^, ^37^ The P53 signaling pathway plays a crucial role in tumorigenesis, as it modulates key cellular functions such as DNA damage repair, cell cycle progression, and apoptosis.^38^, ^39^, ^40^

It was found in our study that LRRC59 was upregulated in BC tissues and this upregulation was associated with poor prognosis, though more clinical studies are required to verify this conclusion. In addition, our study mainly focused on the clinical association between LRRC59 and BC progression and prognosis via the possible regulatory networks, but we are not sure whether LRRC59 regulated BC progression directly through FGF1 or CIP2A pathway. Additionally, we did not use animal models to investigate the role of these biomarkers in BC progression. But at any rate, our results provide a deeper insight into the role of LRRC59 in BC.

However, this study also has some limitations, such as such as only LRRC59 knockdown experiment in cell lines without overexpression, although our in vitro results suggest that LRRC59 knockdown can impair the metastatic potential of BC cells, further studies using animal models are needed to confirm this effect in vivo. Moreover, the downstream regulation mechanism of LRRC59 has not been further studied. These aspects require further experimental validation.

In conclusion, this study preliminarily demonstrated that high expression of LRRC59 was associated with a poor prognosis in BC. Research on prognosis assessment, genes that interact with LRRC59, pathway enrichment and correlations between LRRC59 and the immune microenvironment have upgraded our understanding about the relationship between LRRC59 and BC. Downregulation of LRRC59 expression could inhibit BC cell proliferation and invasion. The above results imply that LRRC59 may be a new target for BC diagnosis and treatment.

## AUTHOR CONTRIBUTIONS

**Peng Zhang:** Data curation (equal); formal analysis (equal); investigation (equal); resources (equal); software (equal); validation (equal); visualization (equal); writing – original draft (equal). **Xiaodu Xie:** Formal analysis (equal); investigation (equal); methodology (equal); software (equal); visualization (equal); writing – original draft (equal). **Chunming Li:** Formal analysis (supporting); investigation (supporting); methodology (supporting); visualization (supporting); writing – original draft (supporting). **Chaohua Zhang:** Methodology (equal); software (equal). **Peihe Liang:** Data curation (lead); funding acquisition (supporting); investigation (lead); methodology (lead); project administration (lead); resources (lead); writing – review and editing (lead).

## FUNDING INFORMATION

This work was supported by the Innovation Program for Chongqing's Overseas Returnees (CX2019146); the High‐level Medical Reserved Personnel Training Project of Chongqing (the fourth batch); and the Research Program of Basic Science and Frontier Technology in Chongqing (cstc2017jcyjAX0435).

## CONFLICT OF INTEREST STATEMENT

The authors declare no potential conflicts of interest with respect to the research, authorship, and/or publication of this article.

## ETHICAL APPROVAL STATEMENT

The study was conducted according to the guidelines of the Declaration of Helsinki, and approved by the ethics committee of the Second Affiliated Hospital of Chongqing Medical University. We declare that written informed consent has been obtained from patients for the use of organizational samples.

## CONSENT FOR PUBLICATION

Not applicable.

## Supporting information


Table S1

Table S2

Table S3

## Data Availability

All the data in the results of this study can be obtained on reasonable request from the corresponding authors.
