# Supplementary material for: LRRC59 serves as a novel biomarker for predicting the progression and prognosis of bladder cancer
Source: Cancer Med. 2023 Sep 14;12(19):19758–76. doi: 10.1002/cam4.6542 (PMC10587936; doi:10.1002/cam4.6542)
Supplement: Supplementary file 1 — Table S1 [file CAM4-12-19758-s001.docx]

**Supplementary Table 1**. Top 10 upregulated and 10 downregulated LRRC59-related DEGs.

| **Gene name** | **Gene id** | **Gene biotype** | **baseMean** | **log2FoldChange** | **lfcSE** | **stat** | **p value** | **p.adj** |
| --- | --- | --- | --- | --- | --- | --- | --- | --- |
| PRSS56 | ENSG00000237412 | Protein coding | 18.70823345 | 5.24519281 | 0.504099331 | 10.4050779 | 2.35064E-25 | 2.34645E-22 |
| KLK5 | ENSG00000167754 | Protein coding | 792.4981918 | 5.216970143 | 0.41858078 | 12.46347275 | 1.18113E-35 | 8.05668E-32 |
| SPANXB1 | ENSG00000227234 | Protein coding | 14.49914419 | 4.222181894 | 0.560934844 | 7.52704515 | 5.19014E-14 | 2.79864E-12 |
| KLK6 | ENSG00000167755 | Protein coding | 757.0336431 | 3.642979286 | 0.333233264 | 10.93221978 | 8.08462E-28 | 1.94635E-24 |
| AC011473.4 | ENSG00000269741 | Protein coding | 36.39826859 | 3.615703811 | 0.446590362 | 8.096242373 | 5.66828E-16 | 5.24855E-14 |
| KLK7 | ENSG00000169035 | Protein coding | 356.11321 | 3.586782452 | 0.389554616 | 9.20739302 | 3.34141E-20 | 1.06011E-17 |
| SPRR2A | ENSG00000241794 | Protein coding | 646.8028822 | 3.501618373 | 0.328987624 | 10.64361732 | 1.86736E-26 | 2.46534E-23 |
| FGF3 | ENSG00000186895 | Protein coding | 2.772319821 | 3.40956516 | 0.697908014 | 4.885407662 | 1.03215E-06 | 8.28453E-06 |
| KRTAP13-2 | ENSG00000182816 | Protein coding | 1.325474826 | 3.333988013 | 0.995634892 | 3.348605036 | 0.000812195 | 0.0028302 |
| ZP4 | ENSG00000116996 | Protein coding | 1.458031662 | 3.268479836 | 0.645481492 | 5.063630605 | 4.11346E-07 | 3.65109E-06 |
| CRTAC1 | ENSG00000095713 | Protein coding | 2697.158313 | -3.770982616 | 0.283896285 | -13.28295864 | 2.90678E-40 | 3.96553E-36 |
| NNAT | ENSG00000053438 | Protein coding | 487.2047601 | -3.026547333 | 0.220740946 | -13.71085605 | 8.74203E-43 | 1.78893E-38 |
| ADGRA1 | ENSG00000197177 | Protein coding | 2.739120842 | -2.842616196 | 0.343737831 | -8.269721695 | 1.34272E-16 | 1.47725E-14 |
| OR1N2 | ENSG00000171501 | Protein coding | 1.527271024 | -2.794818239 | 0.6352252 | -4.399728223 | 1.08387E-05 | 6.47393E-05 |
| BRINP2 | ENSG00000198797 | Protein coding | 23.12002503 | -2.729533957 | 0.298804927 | -9.134835849 | 6.55061E-20 | 1.92875E-17 |
| CLEC2A | ENSG00000188393 | Protein coding | 8.210997173 | -2.55004118 | 0.459953971 | -5.544122547 | 2.95432E-08 | 3.5746E-07 |
| CTSE | ENSG00000196188 | Protein coding | 2854.134274 | -2.528206589 | 0.283678694 | -8.912218794 | 5.00213E-19 | 1.10066E-16 |
| SCRT2 | ENSG00000215397 | Protein coding | 0.799664135 | -2.460446856 | 0.532860385 | -4.617432497 | 3.88517E-06 | 2.65408E-05 |
| SSTR5 | ENSG00000162009 | Protein coding | 12.4281826 | -2.36351706 | 0.344018115 | -6.870327334 | 6.40548E-12 | 2.01531E-10 |
| CRTAC1 | ENSG00000095713 | Protein coding | 2697.158313 | -3.770982616 | 0.283896285 | -13.28295864 | 2.90678E-40 | 3.96553E-36 |
