# Supplementary material for: LRRC59 serves as a novel biomarker for predicting the progression and prognosis of bladder cancer
Source: Cancer Med. 2023 Sep 14;12(19):19758–76. doi: 10.1002/cam4.6542 (PMC10587936; doi:10.1002/cam4.6542)
Supplement: Supplementary file 2 — Table S2 [file CAM4-12-19758-s003.docx]

**Supplementary Table 2**. Gene Ontology pathway functional enrichment for LRRC59-related DEGs.

| **ONTOLOGY** | **ID** | **Description** | **GeneRatio** | **BgRatio** | **p value** | **p adjust** | **q value** | **Gene ID** | **Count** | **z-score** |
| --- | --- | --- | --- | --- | --- | --- | --- | --- | --- | --- |
| BP | GO:0070268 | cornification | 43/730 | 112/18670 | 1.31e-31 | 5.63e-28 | 5.27e-28 | KLK5/SPRR2A/KRT81/KRT6B/PI3/SPRR2G/KRT24/SPRR1B/SPRR2F/CDSN/KRT6C/KRT34/SPRR2E/SPRR2D/KLK13/KLK12/CASP14/SPRR2B/KRT84/KRT9/SPINK6/KRT14/DSC1/KRT75/TGM1/KRT31/KRT32/KRT1/KLK14/KRT35/KRT78/KRT37/KRT71/KRT72/KRT85/KRT38/KRT83/CYP26B1/DSC2/SPRR3/KRT6A/DSG3/KRT16 | 43 | 6.55743852 |
| BP | GO:0031424 | keratinization | 57/730 | 224/18670 | 1.40e-30 | 3.01e-27 | 2.82e-27 | KLK5/SPRR2A/KRTAP13-2/KRT81/KRT6B/PI3/SPRR2G/KRT24/KRTAP3-3/SPRR1B/SPRR2F/CDSN/KRT6C/KRT34/SPRR2E/SPRR2D/KLK13/KRTAP3-1/KLK12/CASP14/SPRR2B/KRT84/KRT9/SPINK6/KRT14/DSC1/LCE3A/KRT75/TGM1/SPRR4/KRTAP19-1/KRT31/KRT32/KRT1/KLK14/KRT35/KRT78/KRT37/KRTAP2-3/KRT71/CNFN/KRT72/KRT85/KRT38/KRT83/CYP26B1/DSC2/SPRR3/KRT6A/DSG3/KRT16/KRTAP10-2/KRTAP5-7/KRTAP5-9/KRTAP5-8/KRTAP5-10/KRTAP5-11 | 57 | 5.96039561 |
| BP | GO:0030216 | keratinocyte differentiation | 65/730 | 305/18670 | 7.53e-30 | 1.08e-26 | 1.01e-26 | KLK5/SPRR2A/KRTAP13-2/KRT81/KRT6B/PI3/S100A7/SPRR2G/KRT24/KRTAP3-3/SPRR1B/SPRR2F/CDSN/KRT6C/KRT34/SPRR2E/SPRR2D/KLK13/KRTAP3-1/KLK12/CASP14/SPRR2B/KRT84/KRT9/SPINK6/EREG/KRT14/DSC1/LCE3A/KRT75/TGM1/SPRR4/KRTAP19-1/KRT31/PRR9/KRT32/KRT1/KLK14/CTSV/KRT35/KRT78/KRT37/KRTAP2-3/KRT71/CNFN/KRT72/KRT85/C1orf68/KRT38/KRT83/CYP26B1/DSC2/SPRR3/SCEL/KRT6A/CD109/DSG3/KRT16/IL20/KRTAP10-2/KRTAP5-7/KRTAP5-9/KRTAP5-8/KRTAP5-10/KRTAP5-11 | 65 | 6.57384093 |
| BP | GO:0009913 | epidermal cell differentiation | 68/730 | 358/18670 | 5.13e-28 | 5.52e-25 | 5.17e-25 | KLK5/SPRR2A/KRTAP13-2/KRT81/KRT6B/PI3/S100A7/SPRR2G/KRT24/KRTAP3-3/SPRR1B/SPRR2F/CDSN/KRT6C/KRT34/SPRR2E/SPRR2D/KLK13/KRTAP3-1/KLK12/CASP14/SPRR2B/KRT84/KRT9/SPINK6/EREG/KRT14/DSC1/LCE3A/KRT75/TGM1/SPRR4/KRTAP19-1/KRT31/PRR9/KRT32/KRT1/KLK14/CTSV/KRT35/KRT78/KRT37/KRTAP2-3/KRT71/CNFN/KRT72/KRT85/C1orf68/KRT38/KRT83/SULT2B1/CYP26B1/DSC2/SPRR3/SCEL/KRT6A/CD109/DSG3/KRT16/IL20/MCOLN3/KRTAP10-2/USH2A/KRTAP5-7/KRTAP5-9/KRTAP5-8/KRTAP5-10/KRTAP5-11 | 68 | 6.54846188 |
| BP | GO:0008544 | epidermis development | 77/730 | 464/18670 | 1.36e-27 | 1.17e-24 | 1.09e-24 | KLK5/KLK7/SPRR2A/KRTAP13-2/KRT81/KRT6B/PI3/S100A7/SPRR2G/KRT24/KRTAP3-3/KRTDAP/SPRR1B/SPRR2F/CDSN/KRT6C/KRT34/SPRR2E/SPRR2D/KLK13/KRTAP3-1/KLK12/CASP14/SPRR2B/KRT84/KRT9/SPINK6/EREG/KRT14/GAL/DSC1/LCE3A/KRT75/TGM1/SPRR4/KRTAP19-1/KRT31/PRR9/KRT32/KRT1/PTHLH/KLK14/LAMC2/CTSV/KRT35/KRT78/KRT37/KRTAP2-3/KRT71/CNFN/KRT72/LAMA3/KRT85/BNC1/C1orf68/KRT38/KRT83/SULT2B1/CYP26B1/DSC2/SPRR3/SCEL/KRT6A/CALML5/CD109/DSG3/KRT16/IL20/MCOLN3/KRTAP10-2/USH2A/KRTAP5-7/SHH/KRTAP5-9/KRTAP5-8/KRTAP5-10/KRTAP5-11 | 77 | 6.95159516 |
| CC | GO:0005882 | intermediate filament | 37/770 | 214/19717 | 2.14e-14 | 5.02e-12 | 4.31e-12 | KRTAP13-2/KRT81/KRT6B/KRT24/NEFL/KRTAP3-3/KRT6C/KRT34/KRTAP3-1/CASP14/KRT84/KRT9/KRT14/KRT75/KRTAP19-1/KRT31/KRT32/KRT1/KRT35/KRT78/KRT37/KRTAP2-3/KRT71/KRT72/KRT85/KRT38/KRT83/KRT6A/KRT16/KRTAP10-2/KRTAP5-7/SYNM/DES/KRTAP5-9/KRTAP5-8/KRTAP5-10/KRTAP5-11 | 37 | 3.45237873 |
| CC | GO:0001533 | cornified envelope | 21/770 | 65/19717 | 2.44e-14 | 5.02e-12 | 4.31e-12 | SPRR2A/PI3/SPRR2G/SPRR1B/SPRR2F/CDSN/SPRR2E/SPRR2D/SPRR2B/DSC1/LCE3A/TGM1/SPRR4/PRR9/KRT1/CNFN/C1orf68/DSC2/SPRR3/SCEL/DSG3 | 21 | 4.58257569 |
| CC | GO:0045095 | keratin filament | 23/770 | 95/19717 | 1.34e-12 | 1.84e-10 | 1.58e-10 | KRT81/KRT6B/KRTAP3-3/KRT6C/KRTAP3-1/CASP14/KRT84/KRT14/KRT75/KRT1/KRT78/KRTAP2-3/KRT71/KRT72/KRT85/KRT83/KRT6A/KRTAP10-2/KRTAP5-7/KRTAP5-9/KRTAP5-8/KRTAP5-10/KRTAP5-11 | 23 | 2.29365855 |
| CC | GO:0045111 | intermediate filament cytoskeleton | 37/770 | 251/19717 | 3.31e-12 | 3.41e-10 | 2.93e-10 | KRTAP13-2/KRT81/KRT6B/KRT24/NEFL/KRTAP3-3/KRT6C/KRT34/KRTAP3-1/CASP14/KRT84/KRT9/KRT14/KRT75/KRTAP19-1/KRT31/KRT32/KRT1/KRT35/KRT78/KRT37/KRTAP2-3/KRT71/KRT72/KRT85/KRT38/KRT83/KRT6A/KRT16/KRTAP10-2/KRTAP5-7/SYNM/DES/KRTAP5-9/KRTAP5-8/KRTAP5-10/KRTAP5-11 | 37 | 3.45237873 |
| CC | GO:0062023 | collagen-containing extracellular matrix | 36/770 | 406/19717 | 4.37e-06 | 3.60e-04 | 3.09e-04 | S100A7/MUC17/L1CAM/KRT1/LAMC2/MMP8/SERPINB12/LAMA3/COCH/COL2A1/F3/SERPINA3/ZG16/LAMA1/SLPI/TNC/CDH2/AMTN/SMOC2/SBSPON/ELANE/TNR/CMA1/COL9A1/BMP7/USH2A/ZP1/COL19A1/MBL2/COL6A5/OGN/AZGP1/MYOC/SHH/AMELX/MUC2 | 36 | 0 |
| MF | GO:0048018 | receptor ligand activity | 59/705 | 482/17697 | 1.33e-14 | 9.35e-12 | 8.26e-12 | FGF3/INSL4/CGA/FGF4/IL36G/CXCL5/PPBP/IL36RN/IL1F10/IL36A/EREG/FGF19/GAL/NRG1/FGF5/PTHLH/AMBN/PPY/SAA1/IL1B/AREG/GAST/GCG/FGF8/CCL7/CXCL11/EDN3/APLN/EPGN/CXCL1/CCL24/IL36B/CSF2/IL20/CGB3/CCL14/CCL15/BMP7/FAM3D/CCL16/COLEC10/BMP5/PROK1/VEGFD/SCGB3A1/NTS/TTR/IL17F/IL25/PTH/IGF2/OGN/C10orf99/TFF1/MSTN/SHH/AMELX/PENK/SST | 59 | 1.43207802 |
| MF | GO:0004252 | serine-type endopeptidase activity | 28/705 | 160/17697 | 3.64e-11 | 1.28e-08 | 1.13e-08 | PRSS56/KLK5/KLK6/KLK7/KLK8/KLK9/KLK10/KLK13/KLK3/KLK12/MMP3/KLK11/KLK14/MMP8/DPP4/PRSS3/F3/KLK2/HPN/TMPRSS11F/TMPRSS11D/ELANE/CMA1/PRSS45P/PRSS54/PRSS48/HPR/LPA | 28 | 2.64575131 |
| MF | GO:0008236 | serine-type peptidase activity | 28/705 | 182/17697 | 8.06e-10 | 1.89e-07 | 1.67e-07 | PRSS56/KLK5/KLK6/KLK7/KLK8/KLK9/KLK10/KLK13/KLK3/KLK12/MMP3/KLK11/KLK14/MMP8/DPP4/PRSS3/F3/KLK2/HPN/TMPRSS11F/TMPRSS11D/ELANE/CMA1/PRSS45P/PRSS54/PRSS48/HPR/LPA | 28 | 2.64575131 |
| MF | GO:0017171 | serine hydrolase activity | 28/705 | 186/17697 | 1.34e-09 | 2.36e-07 | 2.08e-07 | PRSS56/KLK5/KLK6/KLK7/KLK8/KLK9/KLK10/KLK13/KLK3/KLK12/MMP3/KLK11/KLK14/MMP8/DPP4/PRSS3/F3/KLK2/HPN/TMPRSS11F/TMPRSS11D/ELANE/CMA1/PRSS45P/PRSS54/PRSS48/HPR/LPA | 28 | 2.64575131 |
| MF | GO:0004175 | endopeptidase activity | 41/705 | 427/17697 | 1.88e-07 | 2.48e-05 | 2.19e-05 | PRSS56/KLK5/KLK6/KLK7/KLK8/KLK9/KLK10/KLK13/KLK3/KLK12/CASP14/MMP3/KLK11/KLK14/MMP8/MMP20/CTSV/DPP4/PRSS3/F3/MEP1A/PGC/KLK2/HPN/TMPRSS11F/USP26/TRABD2A/TMPRSS11D/ELANE/CMA1/MMEL1/PRSS45P/CAPN9/PRSS54/CAPNS2/PRSS48/ASTL/HPR/LPA/KEL/CTSE | 41 | 2.34260643 |
