# Supplementary material for: LRRC59 serves as a novel biomarker for predicting the progression and prognosis of bladder cancer
Source: Cancer Med. 2023 Sep 14;12(19):19758–76. doi: 10.1002/cam4.6542 (PMC10587936; doi:10.1002/cam4.6542)
Supplement: Supplementary file 3 — Table S3 [file CAM4-12-19758-s002.docx]

**Supplementary Table 3**. KEGG pathway functional enrichment for LRRC59-related DEGs.

| **ONTOLOGY** | **ID** | **Description** | **GeneRatio** | **BgRatio** | **p value** | **p adjust** | **q value** | **Gene ID** | **Count** | **z-score** |
| --- | --- | --- | --- | --- | --- | --- | --- | --- | --- | --- |
| KEGG | hsa04080 | Neuroactive ligand-receptor interaction | 36/312 | 341/8076 | 2.92e-08 | 7.66e-06 | 7.11e-06 | CGA/CHRNA9/GAL/NPFFR2/PPY/GCG/PRSS3/OPRK1/FPR2/GRM3/MC4R/EDN3/HTR1D/APLN/CCKBR/RXFP3/CHRND/GABRA1/P2RX2/ADRB3/CHRNA4/AGTR1/CRHR2/AVPR1B/ADRA2C/CNR2/GIPR/NTS/P2RX1/PTH/P2RX6/CHRM2/RXFP4/PENK/SST/SSTR5 | 36 | -0.333333333 |
| KEGG | hsa00830 | Retinol metabolism | 12/312 | 68/8076 | 9.32e-06 | 0.001 | 0.001 | CYP2B6/RPE65/CYP26B1/DHRS9/RDH16/UGT1A1/UGT1A4/CYP4A22/UGT1A5/UGT1A9/CYP3A5/UGT2B28 | 12 | -0.577350269 |
| KEGG | hsa05150 | Staphylococcus aureus infection | 13/312 | 96/8076 | 7.45e-05 | 0.007 | 0.006 | KRT24/KRT34/KRT9/KRT14/KRT31/KRT32/KRT35/KRT37/FPR2/KRT38/KRT16/FCGR3B/MBL2 | 13 | 3.050851079 |
| KEGG | hsa04060 | Cytokine-cytokine receptor interaction | 25/312 | 295/8076 | 1.75e-04 | 0.011 | 0.011 | IL36G/CXCL5/PPBP/IL36RN/IL1F10/IL31RA/IL36A/IL1B/CCL7/CXCL11/CCR3/CXCL1/CCL24/IL36B/CSF2/IL20/CCL14/CCL15/BMP7/TNFRSF13B/CCL16/BMP5/IL17F/IL25/MSTN | 25 | 1.4 |
| KEGG | hsa04657 | IL-17 signaling pathway | 12/312 | 94/8076 | 2.48e-04 | 0.013 | 0.012 | S100A7/CXCL5/S100A7A/MMP3/MUC5B/IL1B/CCL7/CXCL1/LCN2/CSF2/IL17F/IL25 | 12 | 2.309401077 |
| KEGG | hsa00140 | Steroid hormone biosynthesis | 9/312 | 61/8076 | 5.04e-04 | 0.022 | 0.020 | HSD3B1/SULT2B1/UGT1A1/UGT1A4/UGT1A5/HSD17B2/UGT1A9/CYP3A5/UGT2B28 | 9 | -1.666666667 |
| KEGG | hsa04061 | Viral protein interaction with cytokine and cytokine receptor | 11/312 | 100/8076 | 0.002 | 0.052 | 0.049 | CXCL5/PPBP/CCL7/CXCL11/CCR3/CXCL1/CCL24/IL20/CCL14/CCL15/CCL16 | 11 | 1.507556723 |
| KEGG | hsa00040 | Pentose and glucuronate interconversions | 6/312 | 34/8076 | 0.002 | 0.052 | 0.049 | AKR1B10/UGT1A1/UGT1A4/UGT1A5/UGT1A9/UGT2B28 | 6 | -1.632993162 |
| KEGG | hsa04973 | Carbohydrate digestion and absorption | 7/312 | 47/8076 | 0.002 | 0.052 | 0.049 | SLC5A1/SI/MGAM/HKDC1/AMY2B/CACNA1D/ATP1A2 | 7 | 0.377964473 |
| KEGG | hsa04512 | ECM-receptor interaction | 10/312 | 88/8076 | 0.002 | 0.052 | 0.049 | LAMC2/LAMA3/COL2A1/LAMA1/TNC/TNR/COL9A1/DMP1/COL6A5/DSPP | 10 | 0 |
| KEGG | hsa00830 | Retinol metabolism | 12/312 | 68/8076 | 9.32e-06 | 0.001 | 0.001 | CYP2B6/RPE65/CYP26B1/DHRS9/RDH16/UGT1A1/UGT1A4/CYP4A22/UGT1A5/UGT1A9/CYP3A5/UGT2B28 | 12 | -0.577350269 |
| KEGG | hsa05150 | Staphylococcus aureus infection | 13/312 | 96/8076 | 7.45e-05 | 0.007 | 0.006 | KRT24/KRT34/KRT9/KRT14/KRT31/KRT32/KRT35/KRT37/FPR2/KRT38/KRT16/FCGR3B/MBL2 | 13 | 3.050851079 |
| KEGG | hsa04060 | Cytokine-cytokine receptor interaction | 25/312 | 295/8076 | 1.75e-04 | 0.011 | 0.011 | IL36G/CXCL5/PPBP/IL36RN/IL1F10/IL31RA/IL36A/IL1B/CCL7/CXCL11/CCR3/CXCL1/CCL24/IL36B/CSF2/IL20/CCL14/CCL15/BMP7/TNFRSF13B/CCL16/BMP5/IL17F/IL25/MSTN | 25 | 1.4 |
| KEGG | hsa04657 | IL-17 signaling pathway | 12/312 | 94/8076 | 2.48e-04 | 0.013 | 0.012 | S100A7/CXCL5/S100A7A/MMP3/MUC5B/IL1B/CCL7/CXCL1/LCN2/CSF2/IL17F/IL25 | 12 | 2.309401077 |
| KEGG | hsa00140 | Steroid hormone biosynthesis | 9/312 | 61/8076 | 5.04e-04 | 0.022 | 0.020 | HSD3B1/SULT2B1/UGT1A1/UGT1A4/UGT1A5/HSD17B2/UGT1A9/CYP3A5/UGT2B28 | 9 | -1.666666667 |
